# Pes Anserinus Syndrome Caused by Osteochondroma in Paediatrics: A Case Series Study

**DOI:** 10.2174/1874325001711010397

**Published:** 2017-05-17

**Authors:** Akio Sakamoto, Shuichi Matsuda

**Affiliations:** The Department of Orthopaedic Surgery, Graduate School of Medicine, Kyoto University, Kyoto, Japan

**Keywords:** Paediatrics, Children, Pes anserinus syndrome, Osteochondromas, Tibia, Hereditary multiple exostoses

## Abstract

**Introduction:**

Osteochondroma is a common benign bone tumor, protruding from the underlying normal bone. Osteochondromas can cause pain depending on their location and size. The pes anserinus is located at the proximal medial side of the tibia, where the tendinous insertions of the sartorius, gracilis and semitendinosus muscles collectively attach. Pes anserinus syndrome, or anserine bursitis, is a painful condition of the pes anserinus, and is more common in adults typically with overweight females. The occurrence of pes anserinus syndrome is rare in the paediatric population.

**Results:**

In the current case series, five patients with pes anserinus syndrome due to proximal tibial osteochondroma are reported. Pain was present in all cases, with snapping in one case. The average age of the patients was 13 ± 1.2 years, ranging from 12 to 15 years. Three patients had a single osteochondroma, and two patients had hereditary multiple exostoses. The sizes of the osteochondromas on plain radiographs varied from 0.5 to 2.5 cm, with an average of 1.46 ± 0.83 cm. All lesions characteristically were located at the medial-posterior edge of the proximal tibia. The symptoms resolved in four cases with surgical resection, and persisted in one non-resected patient.

**Conclusion:**

The characteristic location of the osteochondroma causes pes anserinus syndrome, even though the lesion is small. The diagnosis of osteochondroma or pes anserinus syndrome may be overlooked when it occurs in a paediatric population. The symptoms seem to be consistent, and resection of the osteochondroma is necessary for treatment.

## INTRODUCTION

Osteochondroma is the most common benign tumor of bone, and is characterized by an exophytic lesion composed of cortical and medullary bone with an overlying hyaline cartilage cap [1]. Two clinical presentations exist: one is a solitary lesion (solitary osteochondroma) and one involves multiple lesions (hereditary multiple exostoses: HME) [2]. Solitary osteochondroma is a frequent lesion estimated to occur in 1%-2% of individuals [1, 3, 4]. These lesions constitute 20%-50% of benign bone tumors and 10%-15% of all bone tumors [1, 3, 4]. Solitary osteochondroma has a male predilection, ranging from 1:1.6 to 1:3.4 [3]. On the other hand, the estimated prevalence of hereditary multiple exostoses is 1:50,000 to 1:100,000 in Western populations [1, 5, 6].

The pes anserinus is located at the proximal medial side of the tibia, where the tendinous insertions of the sartorius, gracilis and semitendinosus muscles collectively attach [7, 8]. This is approximately 5-7 cm below the antero-medial joint line of the knee [9]. Pain in the pes anserinus region can be diagnosed as pes anserinus syndrome or anserine bursitis [7]. Although the term “anserine bursitis” is commonly used, the structure responsible for the symptoms is not identified, and little is known about the pathology [10]. The diagnosis of pes anserinus syndrome is based on clinical manifestations marked by spontaneous medial knee pain on climbing or descending stairs, tenderness at the pes anserinus insertion and occasional local swelling [7, 8, 11]. The incidence of pes anserinus syndrome is higher in women 50-80 years of age than in younger women or in men [10, 12]. Osteochondroma causing pes anserinus syndrome is a rare condition in the general population [13].

In the current report, five paediatric cases of pes anserinus syndrome due to osteochondroma are described. The article focuses on the clinical character and diagnosis with an emphasis on the paediatric population because the diagnosis of pes anserinus syndrome can be overlooked in this population.

## CASE PRESENTATIONS

A total of five cases of pes anserinus syndrome due to osteochondroma were diagnosed in one boy and four girls with a mean age of 13 ± 1.2 years (range from 12 to 15 years). Pain was present in all patients, and snapping was present in one patient. The sizes of the osteochondromas on plain radiographs varied from 0.5 to 2.5 cm, with an average of 1.46 ± 0.83 cm. The lesions were on the right side in four cases, and on the left side in one case. All lesions characteristically were located at the medial-posterior edge of the proximal tibia. Four lesions were surgically resected, and the symptoms disappeared. One patient did not undergo surgical resection and continued to experience persistent pain at the two-year follow-up. We describe the details of each case below. Clinical data are summarized in Table **1**.

## Case 1

**Fig. (1):**
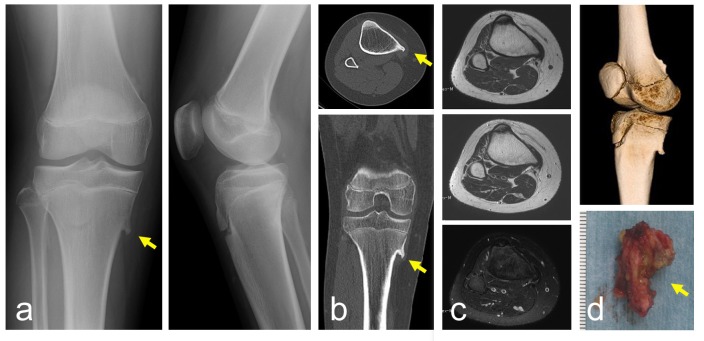
A 12-year-old boy with a solitary osteochondroma. Plain radiographs show a small lesion protruding from the bone, mimicking a bone spur at the proximal tibia (**a**) (left: antero-posterior view, right: lateral view). CT shows that the lesion is located at the medial posterior edge of the tibia (**b**) (top: axial view, bottom: coronal view). MRI shows continuity of bone marrow with the lesion (**c**) (top: T1 weighted image, middle: T2 weighted image, bottom: T2 weighted image with fat-suppression). 3-dimensional reconstruction using CT depicts the lesion (**d**: top). A photograph of a resected specimen (**d**: bottom). (Yellow arrows indicate the tip of the osteochondroma).

A 12-year-old boy presented with pain and a mass on the medial aspect of the right proximal leg. He first noticed symptoms at around 10 years of age. The pain appeared on knee flexion, and was absent when he walked or ran. His height and body weight were 162 cm and 59 kg, respectively. He had no past medical history related to the lesion. There was no history of trauma or injury at the knee. On examination, a non-mobile hard mass was palpable with tenderness at the medical side of the proximal tibia. Swelling was not obvious. Plain radiographs showed a lesion protruding from the bone with continuity to the underlying bone. The size of the lesion was 0.8 cm on a plain radiograph (Fig. **1a**). CT depicted a protuberant bone lesion with continuity to normal bone (Fig. **1b**), but an overlying cartilaginous cap was not obvious. The MRI showed the lesion had continuity to the host bone marrow. There was no obvious inflammation characterized by high signal intensity on a T2 weighted fat-suppression image around the tip of the osteochondroma (Fig. **1c**). For surgical resection, the lesions were approached posterior to the conjoint tendon (Fig. **1d**). While retracting the attachment of the pes anserinus, the lesion was resected using a curved chisel. Histologically, the specimen showed partially sclerotic lamellar bone trabeculae covered by an attenuated hyaline cartilaginous cap undergoing endochondral ossification with fatty marrow, compatible with osteochondroma. There was no evidence of malignancy. Six months after surgery, the symptoms disappeared.

## Case 2

**Fig. (2):**
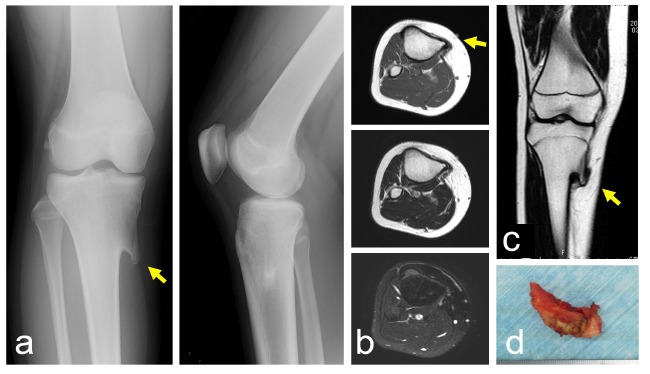
A 13-year-old girl with a solitary osteochondroma. Plain radiographs show a lesion protruding from the proximal tibia (**a**) (left: antero-posterior view, right: lateral view). MRI shows continuity of bone marrow with the lesion in axial (**b**) (top: T1 weighted image, middle: T2 weighted image, bottom: T2 weighted image with fat-suppression) and coronal section (**c**). A photograph of a resected specimen (**d**). (Yellow arrows indicate the tip of the osteochondroma).

A 13-year-old girl presented with pain and swelling on the medial aspect of the proximal leg, which she had noticed for several years. Her height and body weight were 161 cm and 64 kg, respectively. She had no past medical history related to the lesion. There was no history of trauma or injury. On physical examination, the non-mobile hard lesion was palpable with slight tenderness on the medial side of the proximal tibia. Plain radiographs showed a lesion protruding from the bone with continuity to the underlying bone (Fig. **2a**). The size of the lesion was 2.0 cm on a plain radiograph. MRI depicted a protuberant bone lesion with continuity to normal bone. However, an overlying cartilaginous cap or surrounding inflammation was not apparent (Figs. **2b, c**). Surgically, the lesions were approached posterior to the conjoint tendon, and resected using a curved chisel (Fig. **2d**). The lesion was composed of lamellar bone trabeculae with fatty marrow and a covering cartilaginous component, compatible with osteochondroma. There was no evidence of malignancy. Six months after surgery, the preoperative symptoms had disappeared.

## Case 3

**Fig. (3):**
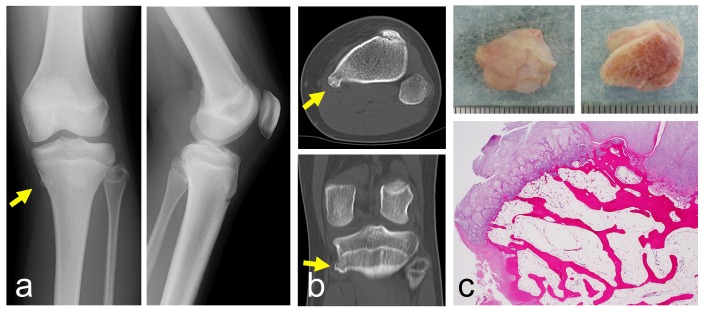
A 13-year-old girl with a small solitary osteochondroma. Plain radiographs show a small osseous lesion on the proximal tibia (**a**) (left: antero-posterior view, right: lateral view). CT shows that the lesion is located at the medial-posterior edge of the proximal tibia (**b**) (top: axial view, bottom: coronal view). A photograph of a resected specimen from the surface (**c**: top-left) and the bottom (**c**: top-right). Histologically, a hyaline cartilaginous cap and underlying lamellar bone trabeculae and fatty marrow are seen (**c**: bottom). (Yellow arrows indicate the tip of the osteochondroma).

A 13-year-old girl had noticed pain for six months when she ran, but no pain while she walked. She also noticed snapping at the medial site of the proximal leg on knee flexion. Her height and body weight were 150cm and 42kg, respectively. She had no past medical history related to the lesion. On physical examination, a small hard lesion was palpable. Tenderness at the medial side of the proximal tibia was observed. The snapping phenomenon was reproduced on knee flexion. Plain radiographs showed a small 0.5 cm osseous lesion on the antero-posterior radiographic view (Fig. **3a**). CT depicted a small protuberant bone lesion with continuity to normal bone (Fig. **3b**). Surgically, the lesions were approached posterior to the conjoint tendon, and resected with a curved chisel while retracting the attachment of the pes anserinus. The lesion was composed of a hyaline cartilaginous cap and underlying lamellar bone trabeculae and fatty marrow (Fig. **3c**). There was no evidence of malignancy. Six months after the surgery, the symptoms had disappeared.

## Case 4

**Fig. (4):**
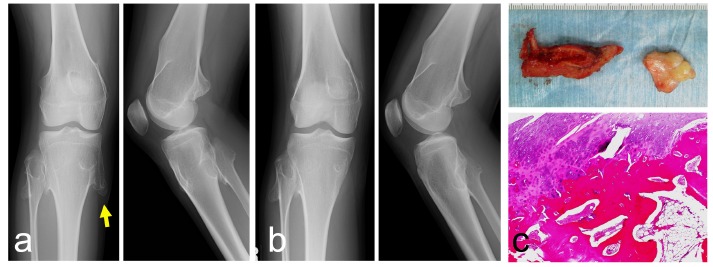
A 12-year-old girl with hereditary multiple exostoses. Plain radiographs show multiple protruding lesions arising from the distal femur, the proximal tibia and the proximal fibula (**a**). Radiographs after the lesion at the pes anserinus is resected (**b**) (left: antero-posterior view, right: lateral view). A photograph of a resected specimen (**c**: top). Histologically, hyaline cartilagninous cap and underlying lamellar bone trabeculae with fatty marrow are seen (**c**: bottom). (A yellow arrow indicates the tip of the osteochondroma causing the pain).

The patient was diagnosed with multiple hereditary exostoses in early elementary school. She had difficulty in fully flexing the right knee because of pain on the medial aspect of the right proximal tibia when she was 12 years old. The symptoms lasted for 2-3 years. She did not have pain while walking. Her height and body weight were 150 cm and 39 kg, respectively. On examination, multiple bony exostoses were palpable on the distal thigh and proximal leg. Tenderness was found only on the lesion of the proximal medial tibia, not at the other lesions. Plain radiographs showed a bony lesion protruding with continuity to the underlying bone in the distal femur and proximal tibia and fibula. The plain radiographic findings permitted a diagnosis of multiple hereditary exostoses (Figs. **4a, b**). The size of the lesion on the medial-posterior proximal tibia was 2.5 cm on a plain radiograph. The resection was performed using a postero-medial approach. The conjoint tendon was retracted anteriorly, and the protuberant lesion was resected using a curved chisel. The lesion was composed of a hyaline cartilage cap and underlying lamellar bone trabeculae with fatty marrow (Fig. **4c**). There was no evidence of malignancy. The medial collateral ligament at the proximal tibia was partially detached. Pain persisted after the operation for about two weeks. Six months after surgery, the preoperative symptoms had disappeared.

## Case 5

**Fig. (5):**
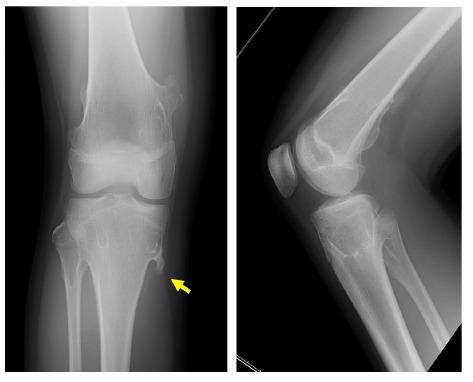
A 15-year-old girl with hereditary multiple exostoses. Plain radiographs show multiple protruding lesions arising from the distal femur, the proximal tibia and the proximal fibula (left: antero-posterior view, right: lateral view). (A yellow arrow indicates the tip of the osteochondroma causing the pain).

The patient had multiple hereditary exostoses from her first decade. When she was 15 years old, she presented with pain on the medial aspect of the right leg. The pain appeared when she changed clothes while sitting on her buttocks and flexing the knee. The pain had been present for six months prior to her visiting a hospital. Her height and body weight were 154 cm and 45 kg, respectively. She had no other specific past history. On examination, multiple bony protuberances from multiple exostoses were palpable at the distal thigh and proximal leg. Tenderness was only found at the medial proximal lesion. Plain radiographs showed a bony protruding lesion on the distal femur and proximal fibula and tibia (Fig. **5**). The size of the protruding lesion on the medial proximal tibia was 1.5 cm on a plain radiograph. Pain appeared occasionally, especially on flexing the knee, but did not disturb activities of daily living. The pain did not require medication, but the symptoms still persisted at the time of the two-year follow-up.

## DISCUSSION

Osteochondromas can cause pain depending on their location and size. Symptomatic lesions usually occur in younger patients, and 75%-80% of such cases are discovered before the age of 20 years [1, 3, 4]. Mechanical effects due to exophytic lesions or bone deformities are associated with the adjacent tendons and ligaments, and lead to restricted joint motion, snapping tendons, and tenosynovitis [1]. Malignant transformation of osteochondroma occurs in approximately 1% of solitary lesions [3]. The prevalence of malignant transformation in hereditary multiple exostoses is thought to be approximately 3%-5% in these patients [1].

A diagnosis of pes anserinus syndrome should be considered when there is spontaneous pain infero-medial to the knee joint [7, 8, 11]. The incidence of pes anserinus syndrome is higher in women who are overweight and 50-80 years old, especially if they also have osteoarthritis of the knees, valgus deformities or pes planus [7, 10, 12]. A second etiology includes osteochondroma, as well as trauma, retraction of posterior thigh muscles, damage to the medial meniscus, infection, and foreign body reaction [10, 14]. The differential diagnosis of pes anserinus syndrome considers lesions with pain infero-medial to the knee joint, such as the medial meniscus; osteoarthritis of the medial compartment of the knee; lumbar radiculopathy; lesions in the medial collateral ligament; and insufficiency fractures [10, 15].

Pes anserinus syndrome is rare in childhood [13], and can be overlooked in a paediatric population with osteochondroma. In the English literature, there is only one case series of pes anserinus syndrome, though pes anserinus syndrome due to osteochondroma is described in a review [10]. The case series includes 9 patients, 6 boys and 3 girls, with an average age of 11 years old, and ranging from 8 to 15 years old. Pain was present in 6 out of 9 cases over the medial aspect of the proximal tibia. Snapping of the pes anserinus tendons or catching of the tendons occurred in 2 out of 9, and locking sensation was seen in 2 out of 9. All patients underwent resection of the lesion, and the symptoms resolved [13]. In the current case series, the average age was 13 years, ranging from 12 to 15. Pain was present in all cases, and one out of 5 patients had snapping. The current clinical findings of age distribution and symptoms were almost the same as in the reported case series. In the current series, resection was performed in 4 out of 5 patients, and the symptoms disappeared. On the other hand, pain persisted in the non-operated patient over a two-year follow-up. It is likely that surgical resection for osteochondroma associated with pes anserinus syndrome is necessary to relieve pain. In making a diagnosis of pes anserinus syndrome due to osteochondroma in this series, the size of the osteochondromas was small, and the lesions were not prominent on the plain radiographs in some cases (Case 3). Careful palpation was required to detect the lesion beneath the pes anserinus in the paediatric population [13].

Plain radiographs generally are not useful in the diagnosis of pes anserinus syndrome. In the majority of the cases, the diagnosis of pes anserinus syndrome was made clinically [8]. The current pes anserinus syndromes were diagnosed by symptoms and physical examination, after the diagnosis of osteochondroma on the plain radiographs. CT and/or MRI were performed in some cases in preparation for surgery for the osteochondroma, rather than for the diagnosis of pes anserinus syndrome. In the diagnosis of pes anserinus syndrome, ultrasonography and MRI do not confirm the clinical diagnosis [10]. However, the characteristic finding of pes anserinus syndrome on ultrasonography includes the thickness of the insertion of the pes anserinus, the presence of fluid collection, and changes in the subcutaneous fat of the medial aspect of the knee. In addition, ultrasonography is useful to exclude the diagnosis of ganglion, meniscal cyst, hemangioma, lipoma, Baker’s cyst, epidermoid cyst, tenosynovitis, hematoma, abscess and pseudoaneurysm [8].

## CONCLUSION

A series of five patients with pes anserinus syndrome due to proximal tibial osteochondromas is described. The lesion is always located at the medial-posterior edge of the proximal tibia. Osteochondromas at this location cause pes anserinus syndrome, even though the lesion is small. All symptoms resolved following surgical excision of the osteochondromas. Pes anserinus syndrome could be overlooked in a paediatric population with osteochondroma, because pes anserinus syndrome is rare in this population.

## Figures and Tables

**Table 1:** Clinical summary of cases of pes anserinus syndrome caused by osteochondroma.

Case No.	Age, Gender	Solitary/HME	Side	Location	Modality	Size (cm) XP	Symptom	Duration	Resection	Final symptom
1	12, M	Solitary	R	Med-post	MRI CT	0.8	Pain	2 y	+	Free
2	13, F	Solitary	R	Med-post	MRI	2.0	Pain	2-3 y	+	Free
3	13, F	Solitary	L	Med-post	CT	0.5	Pain, Snapping	6 mo	+	Free
4	12, F	HME	R	Med-post	XP	2.5	Pain	2-3 y	+	Free
5	15, F	HME	R	Med-post	XP	1.5	Pain	2 y	–	Occasional pain

F: female, L: left, HME: hereditary multiple exostoses, M: male, Med-post: medial-posterior, mo: month, R: right, XP: plain radiograph, y: year.

## LIST OF ABBREVIATIONS

CT: = Computed Tomography
MRI: = Magnetic Resonance Imaging
